# Functional network reorganization and memory impairment in unruptured brain arteriovenous malformations

**DOI:** 10.3389/fnins.2025.1568045

**Published:** 2025-04-09

**Authors:** Anqi Li, Xiaofeng Deng, Kexin Yuan, Yu Chen, Zhipeng Li, Xiaolin Chen, Yuanli Zhao

**Affiliations:** ^1^Department of Neurosurgery, Beijing Tiantan Hospital, Capital Medical University, Beijing, China; ^2^Department of Neurosurgery, Peking Union Medical College Hospital, Chinese Academy of Medical Sciences and Peking Union Medical College, Beijing, China

**Keywords:** working memory, unruptured arteriovenous malformation, neurocognition, resting-state functional magnetic resonance imaging, functional connectivity

## Abstract

**Background:**

Brain arteriovenous malformations (AVMs) are congenital vascular anomalies that can affect cognitive, particularly memory functions. However, the underlying mechanisms of neurocognitive abnormalities in unruptured AVMs remain unclear. This study aimed to explore spontaneous functional network reorganization associated with memory impairment in unruptured AVM patients using resting-state functional MRI (rsfMRI).

**Methods:**

Using rsfMRI data, we compared functional activity and connectivity patterns between 25 AVM patients and healthy controls, including regional homogeneity (ReHo), fractional amplitude of low-frequency fluctuations (fALFF), seed-based functional connectivity (FC), and lesion network mapping. Correlation analysis was performed to clarify the relationship between these parameters and memory performance in AVM patients.

**Results:**

We identified memory-related spontaneous functional network reorganization in AVM patients, particularly involving the somatomotor network (SMN), frontoparietal control network (FPN), and default mode network (DMN). Subgroup analyses based on lesion location (frontal vs. non-frontal) and laterality (left vs. right) revealed location-dependent differences in connectivity reorganization. In particular, left-sided AVMs showed disrupted FC within the SMN, correlated with working memory and executive function, while right-sided and frontal AVMs exhibited more complex patterns involving multiple networks. Moreover, functional disconnection maps indicated that AVM lesions did not directly impair resting-state memory networks.

**Conclusion:**

Patients with unruptured AVMs exhibit resting-state memory network reorganization, which is closely related to the lesion location. These findings highlight the functional network alterations in AVM patients and suggest the potential neural mechanisms underlying memory deficits.

## 1 Introduction

Brain arteriovenous malformations (AVMs) are vascular anomalies characterized by an abnormal connection between feeding arteries and draining veins without the capillary bed (Solomon and Connolly, 2017). Based on clinical presentation, these congenital lesions are primarily divided into two groups: ruptured and unruptured AVMs. Unruptured AVMs can lead to epilepsy, headaches, and focal neurological deficits (Mohr et al., 2014). Neurocognitive abnormalities have been observed in previous studies on unruptured AVMs, with approximately 50–75% of patients demonstrating cognitive impairment in at least one domain (Wenz et al., 1998; Coelho et al., 2019; Itsekson-Hayosh et al., 2024). Among these, working memory, verbal memory, and executive function are the most frequently affected domains, though significant variability exists in the identified risk factors. Frontal and temporal lesions have been reported associated with a higher risk of memory impairment, while executive function appears to be impaired regardless of lesion location (Coelho et al., 2019). These impairments might not meet the criteria for neurological deficits and were therefore often overlooked in clinical research (Coelho et al., 2019; Chen et al., 2020; Berra et al., 2022). Meanwhile, they can still significantly impact patients’ daily activities and overall quality of life. However, the underlying mechanisms of AVM-induced cognitive deficits, particularly memory impairment, remain unclear. One potential explanation is that long-term hemodynamic changes and chronic focal ischemia may alter brain development, ultimately causing disruptions and reorganization within neural networks, which is distinct from cognitive impairments caused by acquired lesions, such as strokes or gliomas (Deng et al., 2023). The process, known as cortical reorganization, involves the redistribution of cognitive functions from affected regions to structurally intact brain areas (Soldozy et al., 2020; Yuan et al., 2025). Previous AVM studies have identified that reorganization of default mode network and control network was associated with working memory and concentration (La Piana et al., 2013). Nevertheless, these studies were limited by insufficient consideration of potential confounding factors, such as epilepsy, which is the second most common clinical manifestation of AVMs and may further influence cognitive outcomes (Bustuchina Vlaicu, 2022; Yuan et al., 2025).

Functional connectivity (FC), measured using resting-state functional MRI (rsfMRI), has been widely applied to explore brain function. Blood oxygen level-dependent (BOLD) signals in rsfMRI enable researchers to examine regional neuroactivity through voxel-based methods, such as regional homogeneity (ReHo) and the fractional amplitude of low-frequency fluctuations (fALFF) (Feng et al., 2021). They could also reveal the interregional connections using seed-based FC approaches. These methods have been employed to investigate the activity changes in various neurological disorders (Fox, 2018). For lesion-induced dysfunctions, characteristic functional alterations have been detected across structurally normal cortical regions and are closely associated with behavioral deficits and functional neuroplasticity (Zhang et al., 2022; Deng et al., 2023). For example, language network remodeling has been observed in unruptured AVMs affecting language areas. Additionally, lesion network mapping (LNM) can quantify the temporal correlation of BOLD signals between the lesion and the rest of the brain, revealing indirect functional disconnections caused by the lesion (Rangus et al., 2024). This approach has been applied to numerous neurological conditions, such as stroke. Since cognition deficits in unruptured AVMs are mostly reversible and are assumed to be associated with chronic functional reorganization, we believe that the above methodologies may help identify resting-state brain networks involved in memory functions affected by unruptured AVMs and offer new insight for the preoperative surgical planning and postoperative rehabilitation therapies (Marshall et al., 2003; Berra et al., 2022).

In this study, we investigated differences in fALFF, ReHo, and FC patterns between AVM patients and healthy controls (HCs) and analyzed the correlation between altered rsfMRI metrics and neurocognitive scores to investigate the spontaneous network reorganization associated with AVM-induced memory impairment in the resting state. We hypothesized that these reorganization patterns vary according to the location and laterality of the AVM nidus. To test this hypothesis, we categorized AVM patients into subgroups based on lesion location and laterality and compared the rsfMRI metrics of these subgroups with those of HCs.

## 2 Materials and methods

### 2.1 Subjects

This study was approved by the Institutional Review Board of Beijing Tiantan Hospital, Capital Medical University (KY 2020-003-01). All procedures adhered to the guidelines of the Declaration of Helsinki. Written informed consent was obtained from all participants. AVM patients were prospectively enrolled between September 2022 and December 2024 based on the following inclusion criteria: (1) age between 18 and 60 years, and (2) AVMs diagnosed based on medical history and radiological findings. The exclusion criteria were: (1) history of intracranial hemorrhage or seizures, (2) prior AVM intervention before data acquisition, (3) poor-quality presurgical MRI, (4) presence of other neurological disorders, and (5) history of drug or alcohol abuse. History of intracranial hemorrhage was identified based on CT and MRI imaging, following the criteria outlined in previous study (Fu et al., 2020). Age-and sex-matched healthy controls (HCs) were recruited from a publicly available dataset using the Hungarian Algorithm (Chaudhry et al., 2013).

### 2.2 Clinical data collection and neurocognitive assessment

Baseline interviews were conducted with AVM patients to collect demographic, including age and sex, and medical history data. MRI scans and neuropsychological assessments were performed on the same day. Neurocognitive assessments included the Montreal Cognitive Assessment Scale (MoCA), the Memory and Executive Screening (MES), the Auditory-Verbal Learning Test-Huashan version (AVLT-H), and the digit span test (DGS) to evaluate the cognitive, especially memory, functions. The MoCA could help detect mild cognitive impairment. The MES reflects executive and memory function, which is independent of reading and writing skills (Guo et al., 2012). The AVLT-H includes short-term delayed recall and long-term delayed recall and represents verbal learning and memory functions (Zhao et al., 2012). The DGS reflects the working memory and attention function (Zuo et al., 2014).

### 2.3 MRI acquisition and preprocessing

MRI of the AVM patients was performed on a 3-Tesla Siemens scanner with a 64-element head–neck coil. The imaging protocol included a three-dimensional T1-weighted sequence and an rs-fMRI sequence with the following parameters: T1 sequence: 176 slices, repetition time (TR) = 2,530 ms, echo time (TE) = 2.02 ms, inversion time (IT) = 1,100 ms, flip angle (FA) = 7°, field of view (FOV) = 256 × 256 mm^2^, matrix size = 256 × 256, voxel size = 1.0 × 1.0 × 1.0 mm^3^; rs-fMRI sequence: 33 slices, TR = 2020 ms, TE = 30.0 ms, FA = 90°, FOV = 224 × 224 mm^2^, matrix size = 64 × 64, voxel size = 3.5 × 3.5 × 3.5 mm^3^. Parameters for the HCs from the Berlin Mind and Brain (BMB) dataset were consistent with those described in previous studies (Rohr et al., 2013; Zuo et al., 2014).

MRI preprocessing was conducted using the fMRIPrep pipeline (version 23.1.4), briefly including head-motion correction, slice-time correction, registration, and spatial normalization to Montreal Neurological Institute (MNI) space (Esteban et al., 2019). Motion, white matter, cerebrospinal fluid, and global signal were regressed out. Quality control involved visual inspection of the fMRIPrep output reports. Data with mean framewise displacement exceeding 0.5 mm were excluded. A 4-mm full-width half-maximum (FWHM) Gaussian kernel was applied during smoothing, following ReHo and fALFF calculations.

Lesions were manually delineated on T1-weighted images using MRIcron (v1.0.20190902) by one neurosurgeon (Dr. Yu Chen) and verified by a senior neurosurgeon (Dr. Xiaolin Chen). Lesion images was normalized into the MNI152 space using the Advanced Normalization Tools (ANTs) and consequently generated the overlapping lesion map using nilearn. Lesion locations were categorized as frontal, temporal, parietal, or occipital lobes. For multi-lobar lesions, categorization was based on the largest affected area. Lesion volumes were calculated using normalized lesion masks in MNI152 space.

### 2.4 Calculation of ReHo and fALFF

ALFF assesses the amplitude of spontaneous low-frequency fluctuations (0.01–0.08 Hz in this study) in BOLD signals, while fALFF is calculated by dividing the ALFF value by the total sum of amplitudes across the entire frequency range. Calculations of ALFF and fALFF were performed using the 3dFSFC tool in the AFNI package. ReHo reflects the temporal homogeneity or similarity of brain activity within a local region of interest. Preprocessed functional MRI data underwent band-pass filtering (0.01–0.08 Hz) before ReHo calculation using the 3dReHo tool in AFNI, based on the Kendall’s coefficient of concordance (KCC), which quantifies the similarity of the time series of each voxel with its 27 nearest neighbors.

Both fALFF and ReHo maps were z-transformed. Voxel-wise comparisons were performed between AVM patients and HCs using permutation tests (10,000 iterations) with Holm-Bonferroni correction (*p* < 0.05). Age and Sex were considered as covariates. Regions showing significant differences were extracted to create threshold maps for further analysis.

### 2.5 Seed-based FC correlation analysis

In the seed-to-brain analysis, regions of significant fALFF and ReHo differences served as seeds. Mean time series within a 4-mm sphere around peak coordinates were extracted. Then we calculated Pearson correlations of time series between the seed and the whole brain regions derived by Schaefer atlas, with lesion-involved regions excluded previously. Fisher-z transformed maps were analyzed using general liner modal (GLM) analysis (FWE-corrected *p* < 0.05), including age and sex as covariates to control for their effects.

In the ROI-to-ROI analysis, we removed the lesion-involved brain regions and extract the mean time series in the rest ROIs. ROIs were derived by the Schaefer atlas and categorized into Yeo’s 7 networks: visual network (VIN), limbic network (LIN), default mode network (DMN), somatomotor network (SMN), dorsal attention network (DAN), ventral attention network (VAN), and frontoparietal control network (FPN) (Schaefer et al., 2018). The Pearson’s correlations of the mean time series between ROI pairs were calculated and underwent Fisher-z transformations to generate correlation matrix for each subject. Correlation matrixes were compared using GLM.

**Figure 1:**
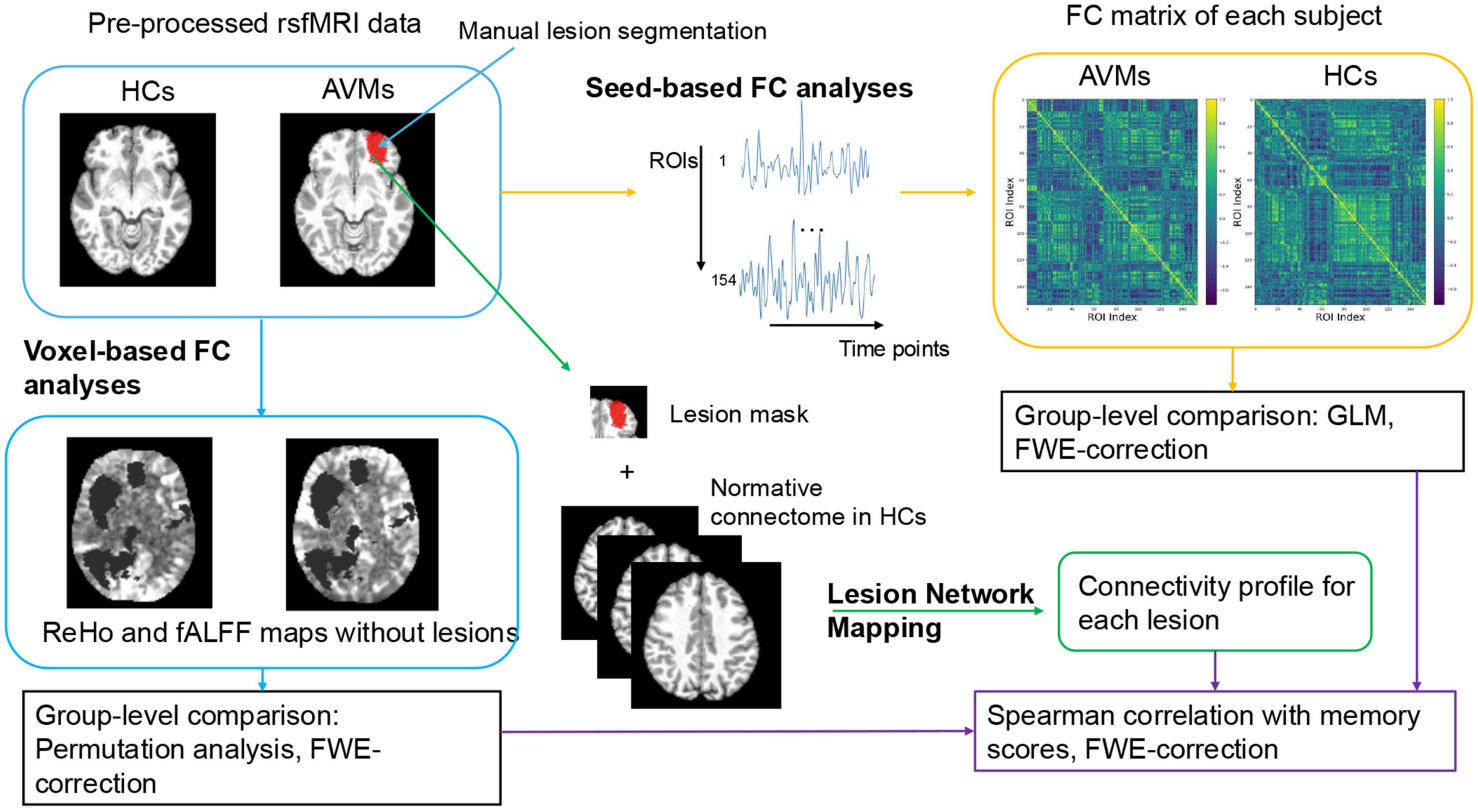
Flowchart of voxel-based and seed-based functional connectivity analyses.

Additionally, LNM provided information about latent dysconnectivity pattern of the lesioned site and was performed following Salvalaggio et al’s. (2020) methodology. Normalized lesion masks were used to create seeds in fMRI data of HCs to compute the Pearson correlations between lesion region and whole brain ROIs. Pearson correlation maps were averaged and derived a functional disconnection map for each lesion. Neuroimaging methods are summarized in Figure 1.

### 2.6 Statistical analysis

Independent-sample t-tests were used for continuous variables, and chi-square tests for categorical variables, with significance set at *p* < 0.05. Statistical analyses for ReHo, fALFF, and FC were detailed in the respective sections. Subgroup analyses were performed based on lesion laterality (left-sided AVM subgroup and right-sided AVM subgroup) and location (frontal AVM subgroup and non-frontal AVM subgroup), with age-and sex-matched HCs in a 1:1 ratio.

Correlation between rs-fMRI metrics and neurocognitive scores were assessed using Spearman tests (FWE-corrected *p* < 0.05), controlling for age, sex, lesion side, location, and lesion volume. Rs-fMRI metrics including threshold ReHo, fALFF maps, and significant different FC metrics were used as independent variables, while neurocognitive scores were used as continuous dependent variables.

## 3 Results

**Figure 2:**
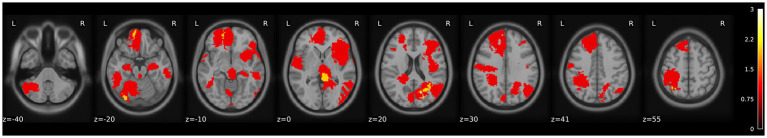
Lesion overlay map of AVM patients. The color bar indicates the number of patients with a lesion in each region.

There was no significant difference in sex ration (13 males in both groups) or age (the median [interquartile range]: 36 [29, 44] years in AVM patients vs. 33 [29, 37] in HCs, *p* = 0.207) between the AVM patients and HCs. Detailed demographic and clinical data of AVM patients are shown in Table 1. Furthermore, AVM patients were categorized based on lesion location (frontal vs. non-frontal) and side (left vs. right). No significant differences were found in demographic or neurocognitive scores between subgroups. Lesion distribution is visualized in Figure 2.

**Table 1:** Baseline characteristics of the AVM patients and subgroups.

	Total	Left AVM subgroup	Right AVM subgroup	*p*	Frontal AVM subgroup	Non-frontal AVM subgroup	*p*
	*n* = 25	*n* = 13	*n* = 12		*n* = 8	*n* = 17	
Age, y	36 [29, 44]	34 [28, 44]	38 [35, 43]	0.400	40 [27, 50]	36 [33, 40]	0.600
Gender, male	13	6	7	1.000	3	10	0.571
Lesion volume, mm^3^	126.6 [70.1, 257.5]	169.4 [85.8, 303.2]	89.8 [18.2, 245.2]	0.314	169.6 [24.4, 578.8]	121.4 [73.9, 243.6]	0.669
MoCA	28 [26, 29]	28 [26, 29]	29 [26, 30]	0.782	28 [24, 29]	28 [27, 29]	0.573
MES	91 [83, 97]	91 [91, 98]	94 [83, 97]	0.956	91 [84, 93]	94 [83, 97]	0.464
AVLT-H	36 [27, 39]	35 [30, 38]	37 [26, 43]	0.892	28 [23, 36]	37 [32, 40]	0.051
DGS-forward	9 [8, 9]	8 [8, 9]	9 [9, 10]	0.100	9 [7, 9]	9 [8, 10]	0.433
DGS-reverse	6 [5, 7]	6 [5, 7]	5 [4, 7]	0.456	6 [4, 7]	6 [5, 7]	0.859

Data are presented as median [IQR] or number.

MoCA, the Montreal Cognitive Assessment Scale; MES, the Memory and Executive Screening; AVLT-H, the Auditory-Verbal Learning Test-Huashan version; DGS, the digit span test.

### 3.1 Regional resting-state activity (ReHo and fALFF) abnormalities

Increased ReHo observed in the left middle temporal gyrus, right calcarine sulcus, and left putamen of AVM patients (*p* < 0.05, FWE corrected). Detailed results are shown in Supplementary Table S1 and Supplementary Figure S1. There was no significant difference of the fALFF values between the AVM patients and HCs. The abnormal regions identified in this study partially align with previous fMRI findings in unruptured AVMs, which were associated with language network reorganization (Deng et al., 2023).

### 3.2 Results of seed-based FC analysis

Altered FC patterns were observed between abnormal ReHo regions and whole-brain seeds in AVM patients (Supplementary Table S2). Decreased FCs were noted between ReHo-identified regions and VAN/DAN ROIs, such as the left supplementary motor area, the left inferior parietal lobule, and the left supramarginal gyrus (*p* < 0.05, FWE corrected).

Large-scale FC abnormality in the non-lesion region was found in AVM patients and AVM subgroups compared to HCs (GLM analysis, FWE corrected *p* < 0.05). Enhanced FC edges were concentrated between the DMN and SMN. Frontal AVMs showed unique FC enhancements within the DMN and more complex whole-brain connectome patterns. Between-group seed-to-seed FC differences are shown in Supplementary Figure S2.

### 3.3 Results of correlation analysis between rsfMRI metrics and neurocognitive scores

No significant correlations were found between ReHo/voxel-to-seed FC alterations and neurocognitive scores. However, some highly activated regions in AVM patients were involved in FCs associated with neurocognitive scores, including MES, AVLT-H, and DGS (Supplementary Table S3).

**Figure 3:**
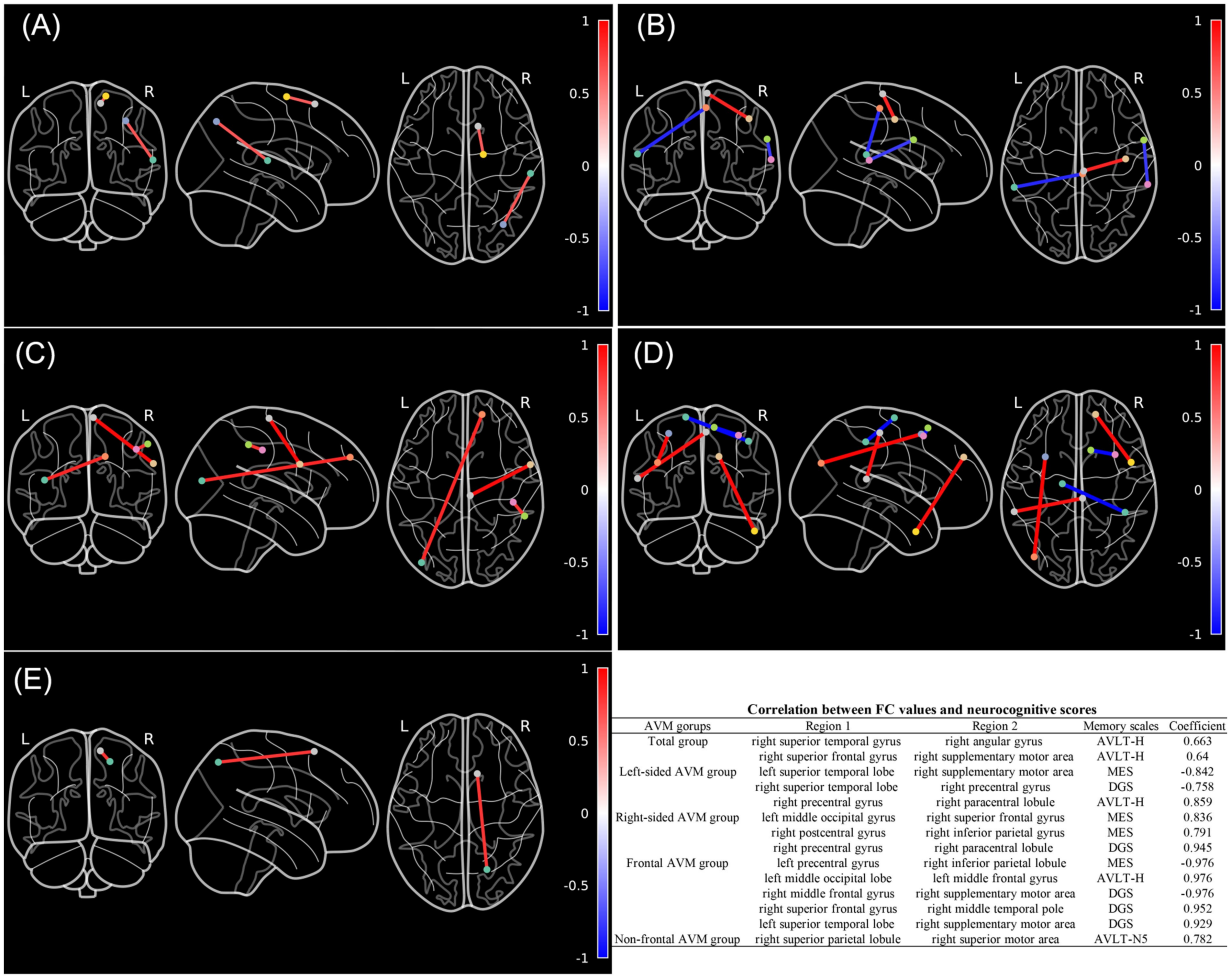
Altered whole brain seed-to-seed functional connectivity patterns associated with memory domains in AVM patients and AVM subgroups compared to HCs. **(A–E)** Illustrate the correlations between altered FCs and neurocognitive scores across different groups, including the entire AVM patient cohort, left-sided AVM patients, right-sided AVM patients, frontal AVM patients, and non-frontal AVM patients. The color bar represents the Spearman correlations coefficients between the FC values and neurocognitive scores. The table provides detailed information on the seed locations, neurocognitive scales, and corresponding Spearman correlation coefficients. (Spearman’s correlation analysis, FWE-corrected *p* < 0.05).

Whole brain ROI-to-ROI FC alterations were found significantly correlated with memory functions and varied by AVM subgroups (Figure 3). In general, increased FCs between regions within the SMN, FPN, and DMN were significantly associated with higher AVLT-H scores. In the left-sided AVMs, reorganized FC within the SMN negatively correlated with MES and DGS-reverse scores. In the right-sided AVMs, we found positive correlations between the MES scores and enhanced FC in VIS, DMN, DAN, and FPN, and between the DGS-reverse scores and enhanced FC within SMN network. In the frontal AVMs, increased FC between the SMN and DAN was negatively associated with the MES score, while enhanced connection among regions in the DMN and FPN was significantly associated with performances of multiple cognitive domains. In non-frontal AVMs, limited FC associations with memory, except for a DMN-DAN FC, which positively correlated with delayed word recall (N5, AVLT-H).

Functional disconnection maps were generated to assess latent disconnections due to AVMs. Spearman correlation test between these patterns and neurocognitive scores revealed that no regions have lesion connectivity significantly associated with memory functions after FWE correction, suggesting that AVM lesions may not directly cut off memory networks.

## 4 Discussion

In this study, we investigated memory dysfunction at both the local and network levels in 25 patients with unruptured AVMs who underwent MRI and detailed neurocognitive assessments. Whole-brain ROI-to-ROI FC analyses identified abnormal connections in the SMN, FPN, and DMN. These disrupted connections were strongly associated with verbal learning and memory. Subgroup analyses further revealed location- and laterality-dependent differences in memory-related FC reorganization: left-sided AVMs exhibited reorganized FC patterns concentrated in the SMN, correlated with working memory and executive function. Right-sided and frontal AVMs displayed more complex reorganization patterns involving multiple networks. LNM analysis suggested that AVM lesions did not have functional connected regions associated with memory performance.

Although unruptured AVM is a intracerebral lesion and affect the local brain tissues, it has different effect on the development of neurological symptoms from other intracranial diseases, such as strokes and brain tumors, due to its congenital nature (Rousseau et al., 2019). Long-term structure and hemodynamic changes may lead to neural plasticity and cause clinical symptoms including seizures, headaches and neurocognitive impairments (Coelho et al., 2019; Rousseau et al., 2019). However, previous studies have largely focused on hemorrhage-related deficits, overlooking patients with impaired cognitive domains but not achieving neurological deficits (Chen et al., 2020). Recent study of Coelho et al. reported a high rate neurocognitive deficits in patients with unruptured AVMs, with memory impairment being the most affected domain (Coelho et al., 2019). Similar findings were observed in patients with unruptured arteriovenous fistulas, who showed significant cognitive improvement following treatments (Itsekson-Hayosh et al., 2024). Moreover, structural neuroimaging studies have provided evidence of reversible white matter alterations linked to cognitive impairment in brain vascular malformations, suggesting a potential relationship between hemodynamic flow states and neurocognitive function (Zeng et al., 2023). However, few studies have specifically explored memory-related functional network alterations in unruptured AVM patients or the relationship between these changes and memory performance. Our study addresses this gap by identifying remodeled FC patterns during resting states and investigating their association with memory function, offering new insights into the neuropathological mechanisms of AVM-induced memory deficits.

We found significantly enhanced FC between SMN, FPN, and DMN regions in AVM patients, which positively correlated with verbal learning and memory. Specifically, these cognition-related FC increases were predominantly detected in the right hemisphere, including connections between the superior temporal lobe and the angular gyrus and between the superior frontal lobe and the supplementary motor area. The association of the angular gyrus and temporal lobe with semantic processing and memory has been widely discussed (Rockland and Graves, 2023). The angular gyrus, often referred to as a “semantic hub,” plays a crucial role in storing multimodal semantic information and engaged positively in studies that examine episodic memory retrieval (Humphreys et al., 2021). Meanwhile, supplementary motor area is considered contributing to the integration of multimodal information into higher-order representations and supporting the verbal working memory together with the frontal region (Cona and Semenza, 2017). These regions compose the frontal–parietal network which collaborate with the DMN regions and may contribute to the information transmission from a non-conscious to conscious level (Caciagli et al., 2023). While connectivity patterns between FPN and SMN are related with the preservation of episodic memory (Hsu et al., 2022). Interestingly, while many studies on memory networks emphasize the left angular gyrus, our findings of enhanced FC involving the right angular gyrus may indicate a “mirror phenomenon” as a result of memory reorganization and reflects compensatory recruitment to maintain memory performance in the AVM patients (Thakral et al., 2017).

Our subgroup analyses showed significant differences in FC reorganization based on AVM laterality and location. Left-sided AVMs exhibited attenuated FC alterations within the SMN, while right-sided AVMs engaged multiple networks to preserve working memory and executive function. Altered FC patterns mainly existed in the right hemisphere. This rightward asymmetry aligns with previous findings that healthy adults rely on the right hemisphere for visuospatial integration, attentional processing, and memory (Wang et al., 2023). AVMs in the right hemisphere may disrupt these functions more extensively, prompting compensatory increases in FC between high-order networks. For frontal AVMs, widespread FC alterations were observed within the SMN, FPN, and DMN, which correlated significantly with the performance of multiple cognition domains. One explanation is that frontal lesions primarily affect local networks that may impair the working memory domain, consequently leading to large-scale FC reorganization in the rest brain for compensation (Parlatini et al., 2017). In contrast, non-frontal AVMs exhibited minimal FC changes related to working memory. Moreover, we observed similar cognitive performance between subgroups, which suggests that the brain’s compensatory mechanisms effectively mitigate functional disruptions.

Our LNM analyses aimed to assess indirect functional disconnection at a whole-brain level but revealed no significant connectivity patterns associated with memory performance. One possible explanation is that unruptured AVMs do not directly impair functional connections between lesion area and the rest of brain but instead induce functional network changes through long-term hemodynamic alterations or chronic ischemia. However, LNM has limitations in detecting subtle white matter disruptions, which might be better captured by structural connectivity analyses (Boes, 2021). Future studies should include these analyses to validate our findings and explore potential white matter pathways involved in AVM-induced cognitive deficits.

This study has several limitations. First, due to the rarity of cerebral AVMs and rigorous exclusion criteria, the sample size was relatively small. Nevertheless, this sample size was sufficient to achieve statistically robust results after strict corrections. Second, BOLD signals may be affected by hemodynamic changes around the AVM nidus. To mitigate this, we excluded lesion areas during data processing. Third, some lesions involved both gray and white matter, with gray matter primarily influencing BOLD signals. This may limit the interpretation of LNM results, particularly for large lesion ROIs. Future research should prioritize structural connectivity analyses to address these challenges and provide a more comprehensive understanding. Fourth, this study did not include cognitive assessment for HCs. We used the identified altered rs-fMRI metrics to explore the relationship between these alterations and memory function in AVM patients but could not evaluate their influences in HCs. Future study containing HCs with neurocognitive assessment results is recommended.

## 5 Conclusion

Memory network reorganization occurs in patients with unruptured AVMs, and this reorganization varies depending on the location and laterality of the lesions. The brain regions recruited into the reorganized memory network primarily involve the SMN, FPN, and DMN, which are associated with working and verbal memory functions. Notably, lesions located in the right hemisphere and frontal areas lead to larger scale of cognitive network alteration and involve a broad range of cognitive domains. LNM analyses suggest that unruptured AVMs do not impair memory functions by directly severing resting-state functional connections between the lesions and other brain regions.

## Data Availability

The datasets presented in this article are not readily available because the data supporting this study’s findings are available from the corresponding author upon reasonable request. The data from the BMB dataset are available following the requirements of CoRR. Requests to access the datasets should be directed to Xiaolin Chen, cxl_bjtth@163.com.
