# Supplementary material for: Functional network reorganization and memory impairment in unruptured brain arteriovenous malformations
Source: Front Neurosci. 2025 Apr 9;19:1568045. doi: 10.3389/fnins.2025.1568045 (PMC12014571; doi:10.3389/fnins.2025.1568045)

Supplementary Material

Table S1. Rigions with significant ReHo changes in the voxel-wise comparison between AVM patents and controls.

Groups	MNI coordinates	Region (AAL)	Size[a]	Peak intensity[b]
AVM group	17.5, -76.5, 7.5	Calcarine_R	1	2.174
	-22.5, 21.5， -6.5	Left_Putamen	10	2.333
	-48.5, -54.5, 1.5	Temporal_Mid_L	1	2.409
Frontal AVM subgroup	-	-	-	-
Non-frontal AVM subgroup	13.5, 57.5, 21.5	Frontal_Sup_2_R	1	2.398
Left-sided AVM group	-	-	-	-
Right-sided AVM group	-38.5, -80.5, 1.5	Occipital_Mid_L	1	2.018

All results were derived using a permutation test with 10000 iterations and Holm-Bonferroni correction ($p<0.05$).

Abbreviations: AAL, anatomical automatic labeling atlas; MNI, Montreal Neurological Institute; ReHo, regional homogeneity.

[a]The cluster size is represented as the number of voxels.

[b]Intensity is the negative logarithm of the voxel-level Bonferroni-corrected p-values.

Figure S1. Significant differences between AVM patients and controls in ReHo analysis. (A) Significant clusters were identified in the left putamen, left middle temporal gyrus, and right calcarine sulcus (from left to right in the figure). Results were derived using a permutation test with 10000 iterations and Holm-Bonferroni correction ($p<0.05$). The color reflects the negative logarithm of the voxel-level Bonferroni-corrected p-values, i.e., $-\log10(p)$. (B) Violin plots illustrate the distribution of ReHo values for voxels with the peak $-\log_{10}(p)$ values within the identified clusters in both groups. T and p were calculated using the permutation test.

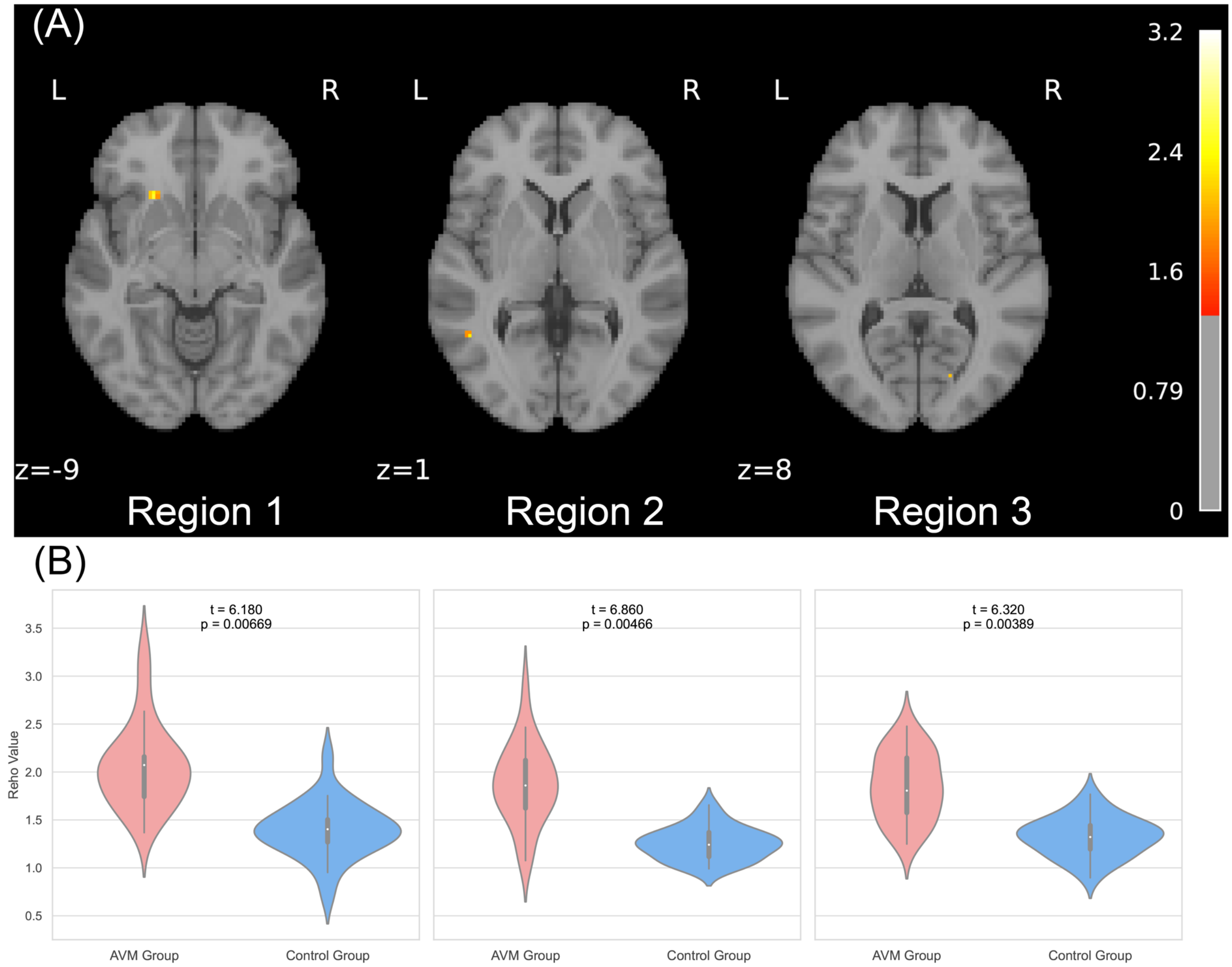

Table S2. Seed-to-brain FC differences between AVM patients and healthy controls.

Seed regions (AAL)	Seed MNI coordinates	Brain regions (AAL)	Brain regions (Schaefer)	t[a]	p
Calcarine_R	(17.5, -76.5,7.5)	Supp_Motor_Area_L	7Networks_LH_SalVentAttn_Med_4	-4.847	0.006
Temporal_Mid_L	(-48.5, -54.5,1.5)	Parietal_Inf_L	7Networks_LH_DorsAttn_Post_6	-4.607	0.015
		SupraMarginal_L	7Networks_LH_SalVentAttn_ParOper_3	-4.467	0.024
Left_Putamen	(-22.5, 21.5, -6.5)	-	-	-	-

All results were derived using general liner modal (GLM) analysis (FWE-corrected $p < 0.05$), including age and sex as covariates.

Abbreviations: AAL, anatomical automatic labeling atlas; Schaefer: Schaefer atlas (400 ROIs); MNI, Montreal Neurological Institute.

[a]T-statistic of GLM model, used to measure the ratio of the estimated regression coefficient to its standard error.

Table S3. Correlation between seed-to-brain FCs and memory scale scores.

Groups	Seed regions (AAL)	Seed MNI coordinates	Brain regions (Schaefer)	Scale	coefficient[a]	P value
AVM patients	Left_Putamen	(-22.5, 21.5, -6.5)	7Networks_RH_Limbic_TempPole_4	AVLT-H	-0.772	<0.001
	Calcarine_R	(17.5, -76.5,7.5)	7Networks_RH_SomMot_32	AVLT-H	-0.698	0.016
			7Networks_RH_SomMot_38	AVLT-H	-0.751	0.002
	Temporal_Mid_L	(-48.5, -54.5,1.5)	7Networks_RH_SomMot_37	AVLT-H	-0.693	0.019
Frontal AVM	-	-	-	-	-	-
Non-frontal AVM	Frontal_Sup_2_R	(13.5, 57.5, 21.5)	7Networks_RH_DorsAttn_Post_1	MES	-0.799	0.018
Left-sided AVM	-	-	-	-	-	-
Right-sided AVM	Occipital_Mid_L	(-38.5, -80.5, 1.5)	7Networks_LH_Vis_20	MES	-0.918	0.010
			7Networks_RH_Default_pCunPCC_7	DGS-reverse	0.927	0.006

Abbreviations: AAL, anatomical automatic labeling atlas; MNI, Montreal Neurological Institute; Schaefer: Schaefer atlas (400 ROIs). MES, the Memory and Executive Screening; AVLT-H, the Auditory-Verbal Learning Test-Huashan version; DGS, the digit span test.

[a]Spearman correlation coefficient (threshold at FWE-corrected $p < 0.05$).

Figure S2 Large-scale FC abnormality in AVM groups compared to HCs. Different colors along the circular periphery of each plot correspond to distinct Yeo brain networks, while the color of the connecting edges reflects the T-values of the significant altered FCs in AVM groups compared to the HCs (GLM analysis, FWE-corrected $p < 0.05$).

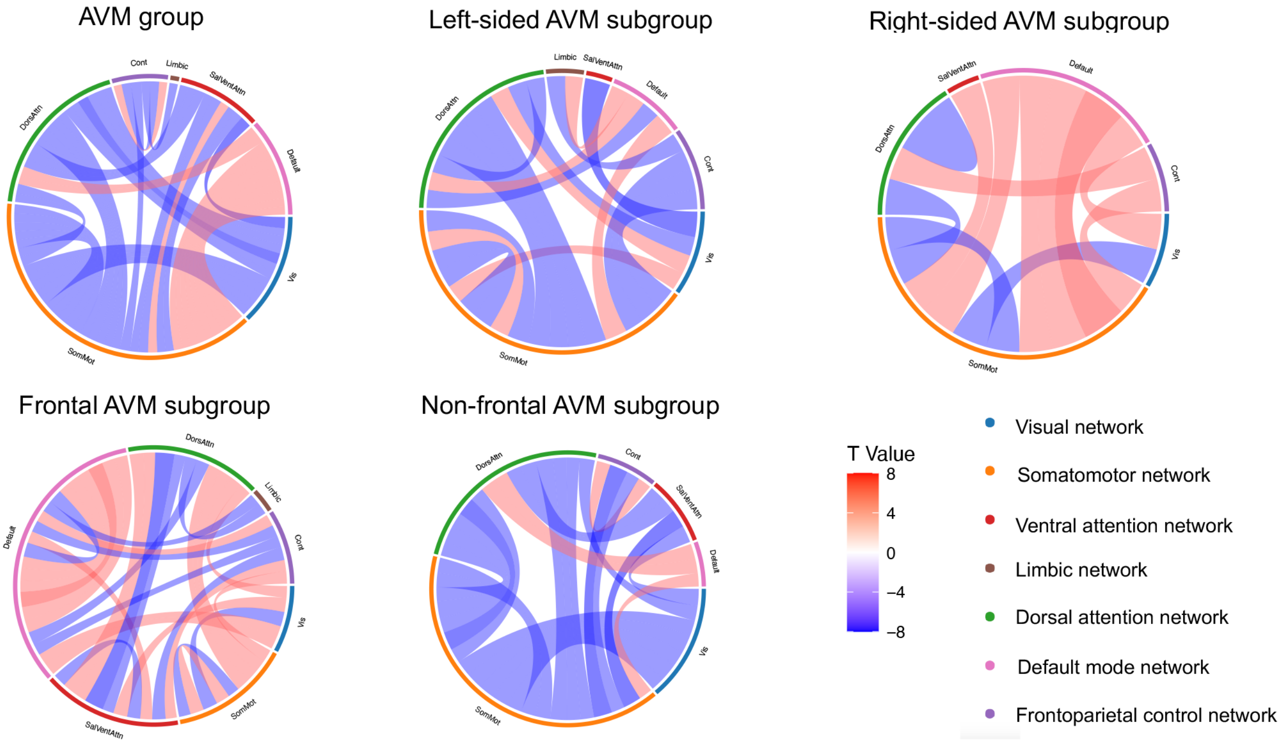

Supplement: Supplementary file 1 [file Data_Sheet_1.PDF]
